# Non-invasive mapping of brown adipose tissue activity with magnetic resonance imaging

**DOI:** 10.1038/s42255-024-01082-z

**Published:** 2024-07-25

**Authors:** Zimeng Cai, Qiaoling Zhong, Yanqiu Feng, Qian Wang, Zuoman Zhang, Cailv Wei, Zhinan Yin, Changhong Liang, Chong Wee Liew, Lawrence Kazak, Aaron M. Cypess, Zaiyi Liu, Kejia Cai

**Affiliations:** 1 Department of Radiology, Guangdong Provincial People’s Hospital (Guangdong Academy of Medical Sciences), Southern Medical University, Guangzhou, China; 2 School of Medicine, South China University of Technology, Guangzhou, China; 3 Guangdong Provincial Key Laboratory of Artificial Intelligence in Medical Image Analysis and Application, Guangzhou, China; 4 Department of Radiology, Guangzhou Women and Children’s Medical Center, Guangzhou Medical University, Guangzhou, China; 5 School of Biomedical Engineering, Southern Medical University, Guangzhou, China; 6 Guangdong Provincial Key Laboratory of Medical Image Processing & Guangdong Province Engineering Laboratory for Medical Imaging and Diagnostic Technology, Southern Medical University, Guangzhou, China; 7 Guangdong-Hong Kong-Macao Greater Bay Area Center for Brain Science and Brain-Inspired Intelligence & Key Laboratory of Mental Health of the Ministry of Education, Southern Medical University, Guangzhou, China; 8 The Biomedical Translational Research Institute, Faculty of Medical Science, Jinan University, Guangzhou, China; 9 Department of Neonatology, Nanfang Hospital, Southern Medical University, Guangzhou, China; 10 School of Medicine, Shenzhen Campus, Sun Yat-sen University, Shenzhen, China; 11 Guangdong Provincial Key Laboratory of Tumor Interventional Diagnosis and Treatment, Zhuhai Institute of Translational Medicine, Zhuhai People’s Hospital Affiliated with Jinan University, Jinan University, Zhuhai, China; 12 Physiology and Biophysics Department, University of Illinois at Chicago, Chicago, IL USA; 13 Rosalind & Morris Goodman Cancer Institute, McGill University, Montreal, Quebec Canada; 14 Department of Biochemistry, McGill University, Montreal, Quebec Canada; 15 Diabetes, Endocrinology, and Obesity Branch, Intramural Research Program, National Institute of Diabetes and Digestive and Kidney Diseases (NIDDK), National Institutes of Health, Bethesda, MD USA; 16 Department of Radiology, University of Illinois at Chicago, Chicago, IL USA; 17 Department of Biomedical Engineering, University of Illinois at Chicago, Chicago, IL USA

**Keywords:** Magnetic resonance imaging, Diagnostic markers, Obesity, Fat metabolism

## Abstract

Thermogenic brown adipose tissue (BAT) has a positive impact on whole-body metabolism. However, in vivo mapping of BAT activity typically relies on techniques involving ionizing radiation, such as [^18^F]fluorodeoxyglucose ([^18^F]FDG) positron emission tomography (PET) and computed tomography (CT). Here we report a noninvasive metabolic magnetic resonance imaging (MRI) approach based on creatine chemical exchange saturation transfer (Cr-CEST) contrast to assess in vivo BAT activity in rodents and humans. In male rats, a single dose of the β_3_-adrenoceptor agonist (CL 316,243) or norepinephrine, as well as cold exposure, triggered a robust elevation of the Cr-CEST MRI signal, which was consistent with the [^18^F]FDG PET and CT data and ^1^H nuclear magnetic resonance measurements of creatine concentration in BAT. We further show that Cr-CEST MRI detects cold-stimulated BAT activation in humans (both males and females) using a 3T clinical scanner, with data-matching results from [^18^F]FDG PET and CT measurements. This study establishes Cr-CEST MRI as a promising noninvasive and radiation-free approach for in vivo mapping of BAT activity.

## Abstract (web summary)

Creatine chemical exchange saturation transfer magnetic resonance imaging is used successfully to measure brown adipose tissue activity in rats and humans, delivering data that are consistent with [^18^F]fluorodeoxyglucose PET and CT measurements.

## Main

Obesity prevalence has markedly increased worldwide in recent decades^1^. In addition to its specific comorbidities, obesity is a major risk factor for other metabolic conditions, such as insulin resistance, type 2 diabetes (T2D) mellitus, cardiovascular diseases and certain types of cancer^2–4^. Obstacles to treating these metabolic diseases are a major global challenge^5,6^. It is noteworthy that activation of thermogenic brown and beige adipocytes^7^ has the potential to be a safe strategy to treat obesity-related metabolic disease^8–10^. Both cold exposure and pharmacological adrenergic receptor activation trigger energy dissipation of existing thermogenic adipocytes in brown adipose tissue (BAT) and convert white adipocytes and adipocyte precursor cells into beige thermogenic adipocytes^11,12^.

Non-shivering thermogenesis through UCP1 is a major mechanism of BAT-mediated energy expenditure^13^. However, additional futile cycles dependent on ATP synthesis and consumption promote energy dissipation in a UCP1-independent manner, such as the futile lipid^14–17^, creatine ^18–21^ and calcium cycles^22^. Creatine is also linked to mitochondrial metabolism and has an important role in the process of mitochondrial oxidative phosphorylation. Therefore, the role of creatine in energy-intensive tissues with a highly variable respiratory rate, and its links to ATP-linked thermogenesis, suggest that creatine could represent a potential biomarker for BAT metabolic function and the diagnosis and treatment of metabolic diseases.

Understanding thermogenic signal pathways and then using them to develop treatments necessitate the development of noninvasive imaging and monitoring methods. Currently, ^18^F-fluorodeoxyglucose ([^18^F]FDG) positron emission tomography (PET) and computed tomography (CT) is the most commonly used method for detecting and quantifying BAT metabolic activity in humans^23,24^. However, radiation exposure and the pharmacodynamic profile of PET tracers limit the use of PET and CT in longitudinal imaging^25^. Furthermore, [^18^F]FDG uptake is fully maintained even when oxygen consumption and adipocyte thermogenesis are diminished, suggesting that increased BAT [^18^F]FDG uptake may occur independently of thermogenesis^26^. On the other hand, magnetic resonance imaging (MRI) displays superior soft tissue contrast and uses only non-ionizing radiation, making it an ideal imaging candidate for longitudinal studies^27^. Most currently available MRI techniques are designed for studying structural information of adipose tissues, such as Dixon’s MRI used for mapping fat-water fraction (FWF). There is no endogenous MRI that is sensitive to the metabolic function of adipose tissues.

Chemical exchange saturation transfer (CEST) MRI is an emerging noninvasive and endogenous metabolic imaging technique that detects tissue metabolites that exchange protons with water, such as those having amine (-NH_2_), amide (-NH), hydroxyl (-OH) or sulfhydryl (-SH) groups. By collecting high-resolution CEST-weighted images at a range of saturation frequency offsets, a Z-spectrum is formed^28,29^. Depending on the abundance of CEST-expressing metabolites in tissue, the exchange rate and the frequency offset of their exchanging protons, CEST MRI is capable of noninvasively imaging proteins^30^, liver glycogen^31^, cartilage glycosaminoglycans^32^ and brain glutamate^33^ with a spatial resolution down to the submillimetre range.

Based on its exchangeable guanidinium protons, creatine CEST (Cr-CEST) MRI has been developed and investigated in several tissues, including brain^34^, tumours^35,36^, heart^37^ and calf muscles^38,39^. As BAT exhibits creatine kinase activity within the same order of magnitude as cardiac or nerve tissue^40^, our goal in this study was to develop Cr-CEST MRI to image the metabolic function of adipose tissues. Cr-CEST MRI for BAT function was validated in animals receiving BAT-activating drugs and then in humans exposed to cold, along with independent PET and CT scans and biochemical measurements. Moreover, the fat signal has been historically treated as an artefact in CEST Z-spectra and researchers have struggled to suppress or remove it^41,42^. We take the opposite and innovative strategy to keep both the fat and Cr-CEST signals in Cr-CEST MRI simultaneously, realizing the combination of fat content and adipocyte metabolic function imaging.

## Results

### Observation and quantification of Cr-CEST from Z-spectra

**Fig. 1:**
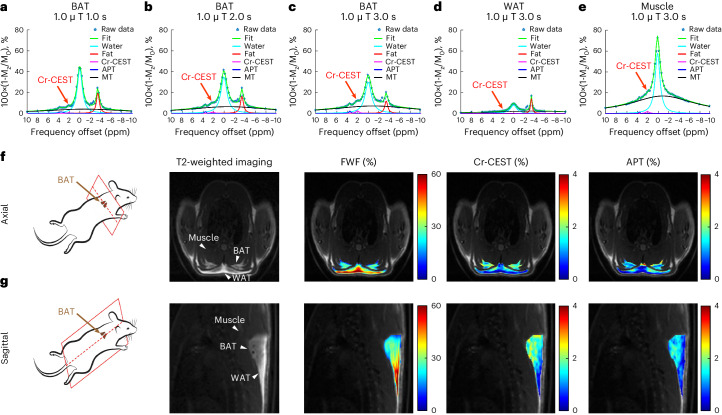
Z-spectral fitting of adipose tissues producing multi-parametric contrasts including Cr-CEST. **a**–**c**, Fitting of the Z-spectra of BAT under different pre-saturation durations: 1.0 μT for 1.0 s (**a**), 2.0 s (**b**) and 3.0 s (**c**). The Cr-CEST peaks (red arrows) could be observed visually. **d**,**e**, Z-spectra of WAT (**d**) and muscle (**e**) under a 1.0 μT saturation for 3.0 s. **f**,**g**, Demonstration of the selection of image slices from the axial (**f**) and sagittal (**g**) views of rats’ interscapular fat depots, representative structural T2-weighted images and the corresponding FWF, Cr-CEST and APT maps obtained from the Z-spectral fitting. Representative image from *n* = 6. In the T2-weighted images, the arrowheads point to the BAT, WAT and muscle regions, respectively.

We acquired Z-spectra, that is, plots of water signal attenuation versus off-resonance saturation frequency, from the rat interscapular fat depot with 1.0 μT saturation power and varied saturation durations (1.0 s, 2.0 s and 3.0 s) on a small-bore horizontal 7T MRI scanner. Relatively low saturation power (1.0 µT) was used for better visualization of any CEST effect from Z-spectra. Z-spectral images (M_z_) with several frequency offsets ranging from −10 to +10 ppm were normalized using a reference image (M_0_) at +300 ppm and corrected for B_0_ inhomogeneities by shifting the dip of water direct saturation to 0 ppm. The reversed pixel-wise raw Z-spectral data (1-M_z_/M_0_, blue stars) were then fitted with multicomponent Lorentzian functions (Fig. 1a–e), corresponding to the peak of water direct saturation (cyan line, centred at 0 ppm), the main methylene fat direct saturation peak (red line, centred at −3.5 ppm), the CEST peak observed at around +2 ppm (Cr-CEST, purple line), the amide proton transfer (APT) effect (blue line, centred at +3.5 ppm) and the broadband semi-solid magnetization transfer (MT) effect (black line, centred between −1 to +1 ppm). The fitting produced several contrast maps, including FWF, Cr-CEST and APT maps (Table 1). The FWF was quantified as the peak amplitude of the methylene fat divided by the sum of the fat and water amplitudes. BAT and white adipose tissue (WAT) in the interscapular fat depot can be visually differentiated in T2_-_weighted images (Fig. 1f,g).

We consistently observed a CEST peak at approximately 2 ppm in the Z-spectra of BAT at all saturation durations. According to the literature, the CEST at 2 ppm peak is attributed to creatine^35,43^ and the 3.5 ppm peak to the APT effect from mobile proteins and peptides^44,45^, which is also visible in BAT Z-spectra. The fitted Cr-CEST peak increased with saturation duration (red arrows, Fig. 1a–c). Therefore, we chose 3.0 s for the rest of the Cr-CEST imaging studies. Compared with BAT, WAT had a lower water signal (Fig. 1d) and muscle tissue had a higher water signal (Fig. 1e), as expected. BAT had higher Cr-CEST and APT signals than WAT (Fig. 1f,g).

### Imaging of BAT activation with Cr-CEST and [^18^F]FDG PET

The selective β_3_-adrenergic receptor agonist CL 316,243 (CL) activated BAT thermogenesis in rodents^46^. To investigate whether Cr-CEST imaging can detect BAT activation, we injected rats with a single intraperitoneal dose of saline or CL (1.0 mg kg^−1^ body weight) and performed dynamic Cr-CEST imaging of the interscapular fat at a 10-min interval for up to 2 h after the injection. For validation, a separate group of rats receiving the same dose of saline or CL was scanned with [^18^F]FDG PET.

**Fig. 2:**
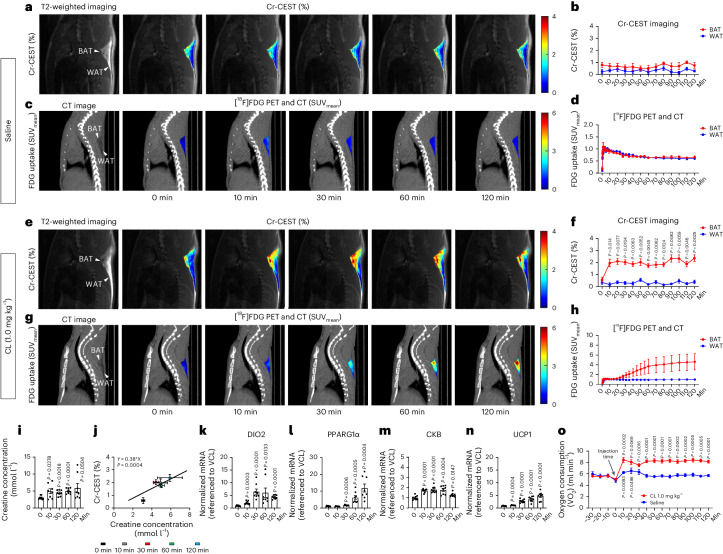
Dynamic Cr-CEST MRI and [^18^F]FDG PET and CT detect the response to saline and the β_3_-adrenergic receptor agonist CL. **a**–**d**, Dynamic Cr-CEST MRI (*n* = 6) and [^18^F]FDG PET and CT (*n* = 4) of interscapular fat depots after intraperitoneal injection with saline in rats: representative images (Cr-CEST % (**a**), FDG uptake SUV (**c**)) and signals (Cr-CEST % (**b**), FDG uptake SUV_mean_ (**d**)). Representative image from *n* = 6 in Cr-CEST MRI and *n* = 4 in [^18^F]FDG PET and CT). **e**–**h**, Dynamic Cr-CEST MRI (*n* = 6) and [^18^F]FDG PET and CT (*n* = 3) of interscapular fat depots after intraperitoneal injection with CL (1.0 mg kg^−1^) in rats: representative images (Cr-CEST % (**e**), FDG uptake SUV_mean_ (**g**)) and signals (Cr-CEST % (**f**), FDG uptake SUV_mean_ (**h**)). Representative image from *n* = 6 in Cr-CEST MRI and *n* = 3 in [^18^F]FDG PET and CT). **i**, ^1^H-NMR analysis of BAT creatine concentration; the creatine concentration of BAT increased after the administration of CL (1.0 mg kg^−1^, *n* = 8, a separate cohort from **f**). **j**, Linear regression analysis of Cr-CEST MRI in relation to creatine concentration (cohorts in **f**,**i**). **k**–**n**, Real-time PCR analysis of thermogenesis-related gene expression due to CL (1.0 mg kg^−1^) in the BAT of rats; all experiments were repeated at least twice with similar results (*n* = 8). **k**, DIO2. **l**, PPARG1α. **m**, CKB. **n**, UCP1. **o**, Dynamic OCR of rats under saline (*n* = 6) or CL (*n* = 8) stimulation. Data are presented as the mean ± s.e.m. Statistical analysis was performed using two-tailed paired (**f**,**o**) and unpaired (**i**,**k**–**n**) Student’s *t*-tests; each time point (10–120 min) was compared to 0 min (**f**,**i**,**k**–**n**) and −10 min (**o**), respectively. In the T2-weighted and CT images, the arrowheads indicate the BAT and WAT regions, respectively. Source data

For rats receiving only saline, the dynamic Cr-CEST signals of BAT (0.80 ± 0.21%) and WAT (0.25 ± 0.16%) were relatively stable after the injection with saline (Fig. 2a,b). There was no difference in FDG uptake between BAT and WAT due to saline; the mean standard uptake value (SUV_mean_) of both BAT and WAT increased rapidly after FDG injection and then gradually decreased to a relatively stable value (Fig. 2c,d), reflecting the pharmacodynamics of [^18^F]FDG.

**Extended Data Fig. 1:**
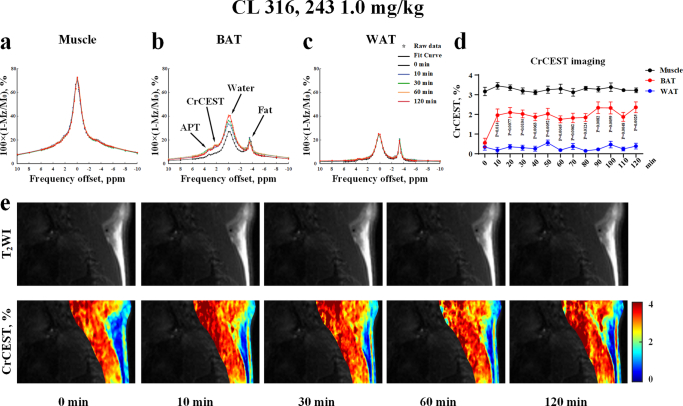
Representative Z-spectra (a-c), CrCEST (d), and CrCEST maps (e) under CL 316, 243 stimulation for 2 hours in rats. Representative image (from n = 6). Data are presented as means ± s.e.m, n = 6. When activated, the CrCEST signal of BAT increased, accompanied by the increase of water signal and the decrease of fat signal. Statistical analysis was performed using two-tailed paired Student’s t-tests, and each time point (10–120 min) was compared to 0 min (d), respectively. In Z-spectra, arrows were pointed to the water, fat, CrCEST, and APT peaks, respectively. Note that the area containing lung cavity is not shown due to sparse signal and artifacts. Source data

We can directly observe from the Z-spectrum that only BAT showed obvious changes after CL administration, that is, an increase in the Cr-CEST signal, accompanied by an increase in the water signal and a decrease in the fat signal, indicating a decrease in FWF during BAT activation (Extended Data Fig. 1). What is more, we did not observe any significant changes in WAT using either Cr-CEST MRI (Fig. 2e,f) or PET and CT imaging (Fig. 2g,h). In contrast, the Cr-CEST from BAT increased significantly within 20 min from 0.56 ± 0.23% to 1.96 ± 0.31% and plateaued for the remaining 2 h after the injection (Fig. 2e,f). Similarly, the FDG uptake in BAT over 2 h in the [^18^F]FDG PET and CT experiments increased continuously (Fig. 2g,h).

Next, a separate group of rats was used to examine CL-mediated creatine changes with ^1^H-nuclear magnetic resonance (^1^H-NMR). The creatine concentration in BAT measured with ^1^H-NMR showed a significant increase within 20 min after the injection with CL (Fig. 2i) and was well correlated with Cr-CEST MRI across different time points (Fig. 2j). CL administration triggered an increase in thermogenic gene expression, including *Dio2*, *Ppargc1α*, *Ckb* and *Ucp1* (Fig. 2k–n). In addition, the injection with CL triggered a rise in the oxygen consumption of rats within 20 min, which was sustained for 2 h, and matched well with the Cr-CEST results (Fig. 2f,o), indicating that Cr-CEST is closely related to pharmacological BAT activation.

### Effect of norepinephrine activation on BAT detection using Cr-CEST and [^18^F]FDG PET

**Fig. 3:**
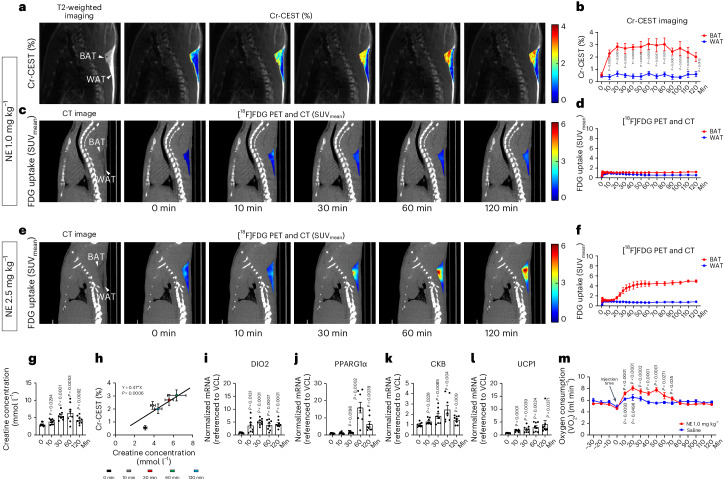
Dynamic Cr-CEST MRI detects the response to NE. **a**–**d**, Dynamic Cr-CEST MRI (*n* = 6) and [^18^F]FDG PET and CT (*n* = 4) of interscapular fat depots after intraperitoneal injection with NE (1.0 mg kg^−1^) in rats: representative images (Cr-CEST % (**a**), FDG uptake SUV_mean_ (**c**)) and signals (Cr-CEST % (**b**), FDG uptake SUV_mean_ (**d**)). Representative image from *n* = 6 in Cr-CEST MRI and *n* = 4 in [^18^F]FDG PET and CT. **e**,**f**, Dynamic [^18^F]FDG PET and CT (*n* = 3) of interscapular fat depots after intraperitoneal injection with NE (1.0 mg kg^−1^) in rats: representative images (FDG uptake SUV_mean_ (**e**)) and signals (FDG uptake SUV_mean_ (**f**)). **g**, ^1^H-NMR analysis of BAT creatine concentration. The creatine concentration of BAT increased after the administration of NE (1.0 mg kg^−1^, *n* = 8, separate cohort from **b**). **h**, Linear regression analysis of Cr-CEST MRI in relation to creatine concentration (cohorts in **b**,**g**). **i**–**l**, Real-time PCR analysis of thermogenesis-related gene expression due to NE (1.0 mg kg^−1^) in the BAT of rats; all experiments were repeated at least twice with similar results (*n* = 8). **m**, Dynamic OCR of rats under saline (*n* = 6) or NE (*n* = 8) stimulation. Data are presented as the mean ± s.e.m. The statistical analysis was performed using two-tailed paired (**b**,**m**) and unpaired (**g**,**i**–**l**) Student’s *t*-tests; each time point (10–120 min) was compared to 0 min (**b**,**g**,**i**–**l**) and −10 min (**m**), respectively. In the T2-weighted and CT images, the arrowheads indicate the BAT and WAT regions, respectively. Source data

**Extended Data Fig. 2:**
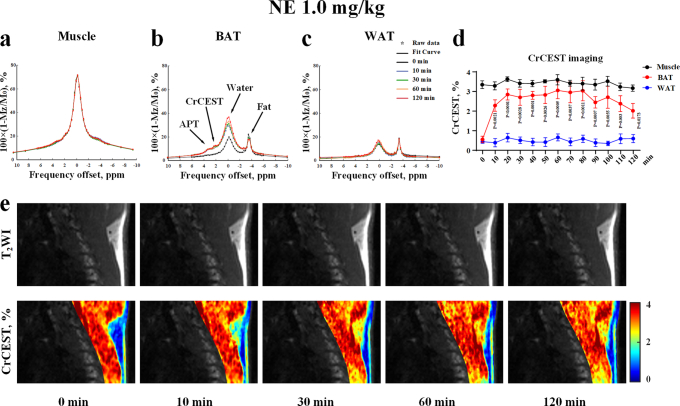
Representative Z-spectra (a-c), CrCEST (d), and CrCEST maps (e) under NE stimulation for 2 hours in rats. Representative image (from n = 6). Data are presented as means ± s.e.m, n = 6. When activated, the CrCEST signal of BAT increased, accompanied by the increase of water signal and the decrease of fat signal. Statistical analysis was performed using two-tailed paired Student’s t-tests, and each time point (10–120 min) was compared to 0 min (d) respectively. In Z-spectra, arrows were pointed to the water, fat, CrCEST, and APT peaks, respectively. Note that the area containing lung cavity is not shown due to sparse signal and artifacts. Source data

Unlike CL, which stimulates rodent BAT activity via the selective β_3_-adrenergic receptor agonist, norepinephrine (NE) targets both α-adrenergic and β-adrenergic receptors^47^. We tested whether NE-stimulated BAT activation could also be detected with Cr-CEST MRI. After NE administration (1.0 mg kg^−1^), we directly observed from the Z-spectrum that only BAT showed obvious changes (Extended Data Fig. 2), which is consistent with the previous stimulation with CL (Extended Data Fig. 1). Furthermore, the Cr-CEST from BAT increased significantly within 20 min from 0.56 ± 0.16% to 2.29 ± 0.29% and was significantly high for 2 h after the injection (Fig. 3a,b). Similarly, the creatine concentration in BAT, measured with ^1^H-NMR, significantly increased within 20 min after NE administration (Fig. 3g) and correlated well with Cr-CEST at different time points (Fig. 3h). Like CL, NE treatment increased the mRNA expression of thermogenic genes, such as *Dio2*, *Ppargc1α*, *Ckb* and *Ucp1* (Fig. 3i–l). In addition, the trend of oxygen consumption rate (OCR) in rats after NE stimulation agreed with the Cr-CEST results; both increased within 20 min and gradually decreased in the later stage (Fig. 3b,m). The transient nature of NE is probably due to its metabolism in vivo, and possibly because of its lower lipolytic action compared to CL^48^.

However, in the PET and CT experiment, the SUV_mean_ value of BAT was low for 2 h after the injection with 1.0 mg kg^−1^ NE (Fig. 3c,d). Only by increasing the NE dose to 2.5 mg kg^−1^, the SUV_mean_ value of BAT significantly increased and lasted for 2 h (Fig. 3e,f), indicating the higher sensitivity of Cr-CEST MRI compared with [^18^F]FDG PET and CT in detecting BAT activation.

**Extended Data Fig. 3:**
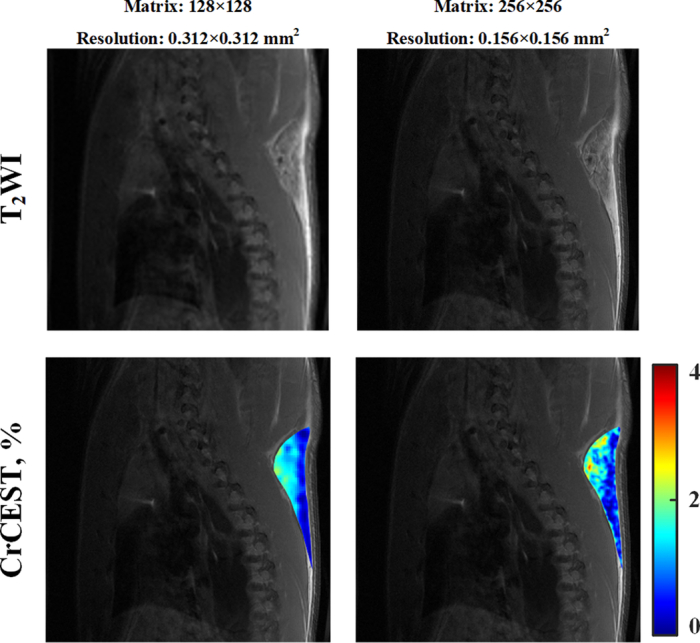
CrCEST MRI at different spatial resolutions. Increasing the spatial resolution helps to reduce the partial volume effect, delineate tissue boundaries, and therefore differentiate different types of adipose tissues. However, a longer scan time is required (8 mins vs. 16 mins). Representative image (from n = 3). Source data

**Extended Data Fig. 4:**
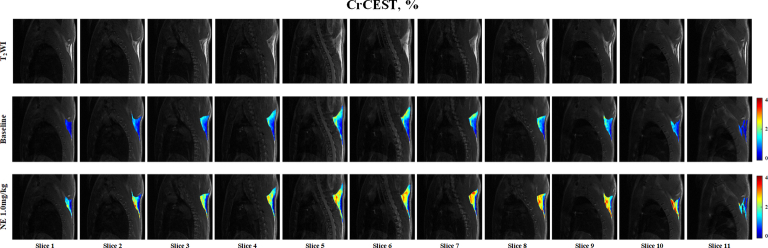
Multi-slice CrCEST imaging of the entire interscapular fat depot 30-min post-injection of NE. The total scanning time is 14 mins. Representative image (from n = 3).

Improving the resolution of Cr-CEST imaging (from an in-plane resolution of 0.312 × 0.312 mm^2^ to 0.156 × 0.156 mm^2^), despite a longer imaging time (from 8 to 16 min), reduced the partial volume effect and clearly delineated the boundaries between BAT and WAT (Extended Data Fig. 3). Multi-slice Cr-CEST MRI covering the entire rat interscapular fat depot were also demonstrated 30 min after NE stimulation (Extended Data Fig. 4).

### Cr-CEST detects BAT adrenergic activation in cold-exposed rats

**Fig. 4:**
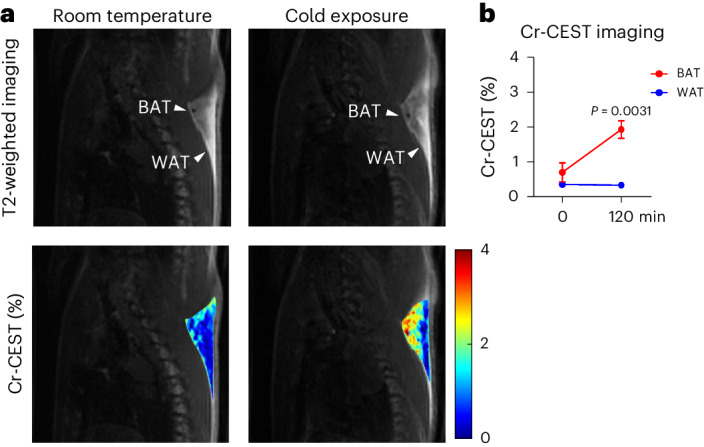
Cr-CEST MRI detects BAT adrenergic activation in cold-exposed rats. **a**, Quantitative maps of BAT Cr-CEST at room temperature and under cold exposure. **b**, BAT Cr-CEST increased significantly after a 2-h cold exposure, while WAT Cr-CEST showed no significant difference before and after cold exposure. Representative image from *n* = 7. Data were acquired from Cr-CEST imaging (*n* = 7). Data are presented as the mean ± s.e.m. The statistical analysis was performed using two-tailed paired Student’s *t*-tests (**b**). In the T2-weighted images, the arrowheads indicate the BAT and WAT regions, respectively. Source data

**Extended Data Fig. 5:**
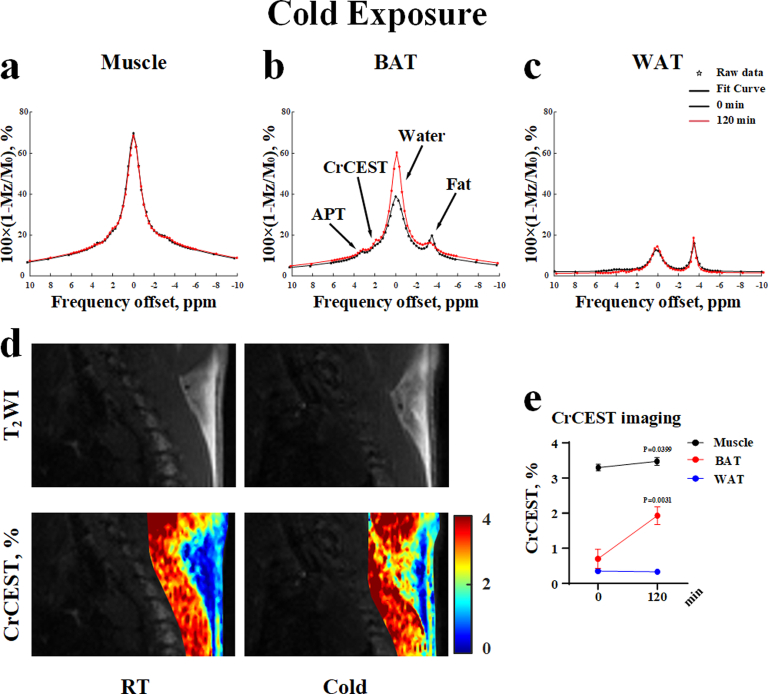
Representative Z-spectra (a-c), CrCEST maps (d), and CrCEST (e) under room temperature (RT, 0 min) and 2-hour cold exposure in rats. Representative image (from n = 7). Data are presented as means ± s.e.m, n = 7. When activated, the CrCEST signal of BAT increased, accompanied by the increase of water signal and the decrease of fat signal. Statistical analysis was performed using two-tailed paired Student’s t-tests (d). In Z-spectra, arrows were pointed to the water, fat, CrCEST, and APT peaks, respectively. Note that the area containing lung cavity is not shown due to sparse signal and artifacts. Source data

Cold exposure activates BAT thermogenesis^49^. Individual rats were placed in precooled cages without bedding at 4 °C for 2 h. The results of the BAT Cr-CEST signal at room temperature and after 2 h of acute cold exposure (4 °C) are shown in Fig. 4 and Extended Data Fig. 5. In response to cold exposure, the Z-spectrum of muscle showed a slightly increased Cr-CEST signal, while BAT showed significantly increased water and Cr-CEST signal and decreased fat signal; WAT showed no difference before and after cold exposure (Extended Data Fig. 5). The dynamic Cr-CEST in rat muscle was stable due to drugs (Extended Data Figs. 1 and 2); it showed a small increase after cold exposure (Extended Data Fig. 5), indicating that cold exposure in rats induced a small thermogenic effect in muscle also. BAT Cr-CEST increased significantly from 0.71 ± 0.27% at room temperature to 1.95 ± 0.25% after exposure to cold (Fig. 4). In contrast, WAT showed no statistically significant differences in Cr-CEST signal between room temperature (0.36 ± 0.06%) and cold (0.34 ± 0.06%) (Fig. 4).

### Cr-CEST MRI detects cold-activated BAT in humans

**Fig. 5:**
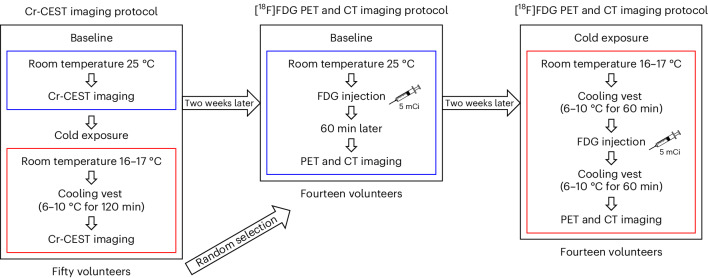
Flowchart of the human studies at room temperature (baseline) and after cold exposure. Fourteen individuals were randomly selected for PET and CT imaging from 50 healthy volunteers selected for Cr-CEST imaging.

**Extended Data Fig. 6:**
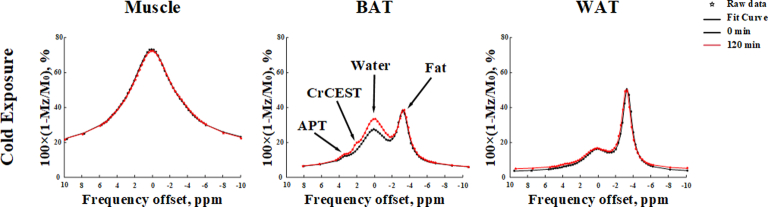
Z-spectra under room temperature (RT, 0 min) and 2-hour cold exposure in humans. The CrCEST and water signals of BAT Z-spectra increased significantly after 2 hours mild-cold exposure. In Z-spectra, arrows were pointed to the water, fat, CrCEST, and APT peaks, respectively.

To evaluate the feasibility of Cr-CEST imaging of human BAT in a clinical 3T MRI, we performed Cr-CEST MRI and [^18^F]FDG PET and CT imaging in healthy individuals exposed to mild cold (Fig. 5). Fifty healthy volunteers (27 men and 23 women) underwent Cr-CEST imaging; from this group, 14 randomly selected individuals (7 men and 7 women) also underwent [^18^F]FDG PET and CT imaging. After being exposed to a mild cold temperature for 2 h, the human Z-spectra data showed that only the water and Cr-CEST peaks had significantly increased signals (Extended Data Fig. 6). Unfortunately, for the human studies using a 3T clinical scanner, because of the broad water direct saturation spectrum affecting the nearby Cr-CEST peak (Extended Data Fig. 6), it was hard to separate the Cr-CEST peak from the water peak. Therefore, fitting the muscle Z-spectrum for consistent and comparable Cr-CEST to the adipose tissues (BAT and WAT) was challenging. Such a task can be better achieved in rats under ultrahigh field 7T MRI, where the water and Cr-CEST peaks are more distinctive (Extended Data Figs. 1, 2 and 5). It is worth noting that the fat peak in human (Extended Data Fig. 6) BAT did not change as much as the fat peak of rats after cold exposure (Extended Data Fig. 5) probably because of the different cold exposure conditions in humans (RT of 16–17 °C and a 6–10 °C cooling vest for 2 h) compared with rats (2 h in climate-controlled cold rooms at 4 °C).

**Fig. 6:**
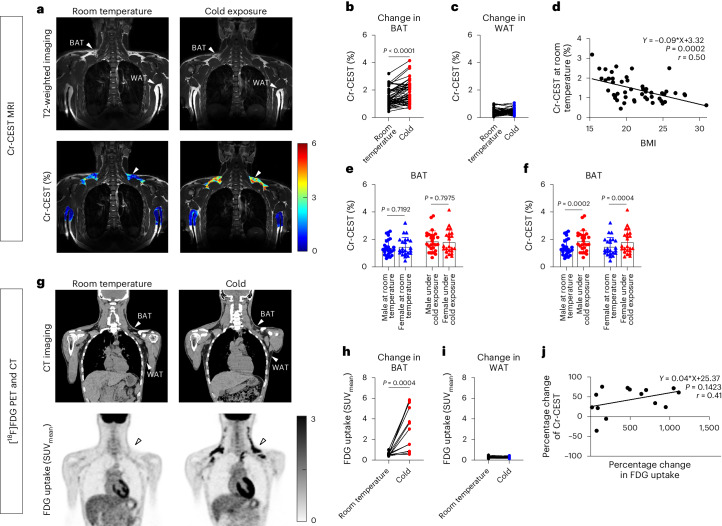
Cr-CEST MRI detects BAT adrenergic activation in cold-exposed humans. **a**, Representative Cr-CEST maps for both the BAT and WAT regions in humans before and after cold exposure. **b**, BAT Cr-CEST increased significantly after a 2-h cold exposure. **c**, WAT Cr-CEST showed no difference before and after cold exposure. **d**, Linear regression analysis of resting-state Cr-CEST MRI in relation to BMI. **e**, Under the same experimental conditions (room temperature or cold exposure), there was no statistical difference in BAT Cr-CEST between male (*n* = 27) and female (*n* = 23) volunteers. **f**, BAT Cr-CEST in both male and female volunteers showed the same statistical power before and after cold exposure. **g**, [^18^F]FDG PET and CT imaging of interscapular fat depots at room temperature and under cold exposure. **h**,**i**, Differences in BAT (**h**) and WAT (**i**) FDG uptake before and after cold exposure. **j**, Linear regression analysis on the percentage change of Cr-CEST in relation to FDG uptake using [^18^F]FDG PET in volunteers who underwent both Cr-CEST and [^18^F]FDG PET imaging (*n* = 14). Representative image from *n* = 50 in Cr-CEST MRI and *n* = 14 in [^18^F]FDG PET and CT. Data were acquired from Cr-CEST (*n* = 50) and PET and CT imaging (*n* = 14). The statistical analysis was performed using two-tailed paired (**b**,**f**,**h**) and unpaired (**e**) Student’s *t*-tests. In the T2-weighted and CT images, the arrowheads indicate the BAT and WAT regions, respectively. Source data

Cr-CEST in the supraclavicular BAT increased from 1.44 ± 0.09% at room temperature to 1.84 ± 0.11% after cold exposure (Fig. 6a,b; *P* < 0.001). In contrast, the subcutaneous abdominal WAT Cr-CEST was the same before (0.41 ± 0.03%) and after (0.44 ± 0.03%) cold exposure (Fig. 6c). Cr-CEST correlates with body mass index (BMI) at RT; this may explain the variations in Cr-CEST at room temperature (Fig. 6d). The BAT and WAT FDG uptake in humans, as measured using the [^18^F]FDG PET SUV_mean_, was consistent with the Cr-CEST MRI (Fig. 6g–i). However, because of the unmatched sample sizes of Cr-CEST (*n* = 50) and [^18^F]FDG PET (*n* = 14), only a moderate correlation was found between Cr-CEST MRI and [^18^F]FDG PET (Fig. 6j). In addition, we found no differences in Cr-CEST and its changes between men and women (Fig. 6e–f).

### The influence of temperature on the Cr-CEST signal

**Extended Data Fig. 7:**
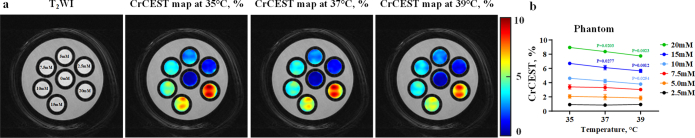
CrCEST MRI of creatine solutions at different temperatures. Relationship between CrCEST and temperature for different creatine concentrations (2.5 mM, 5.0 mM, 7.5 mM, 10 mM, 15 mM, and 20 mM), n = 3. (**a**) T_2_WI, and the CrCEST maps at different temperatures (35 °C, 37 °C, and 39 °C respectively). (**b**) When the concentration of creatine was equal to or higher than 10 mM, the CrCEST signal showed a tendency to gradually decrease from 35 °C to 39 °C. Representative image (from n = 3). Data are presented as means ± s.d. Statistical analysis was performed using two-tailed paired Student’s t-tests. Each temperature (37 and 39 °C) was compared to 35 °C. Source data

We investigated the effect of temperature on Cr-CEST imaging using in vitro phantom experiments. Phantoms (*n* = 3 sets) contain vials of creatine with concentrations of 2.5 mM, 5.0 mM, 7.5 mM, 10 mM, 15 mM and 20 mM at pH 7.0. By heating the phantoms with a hot-water pad, we investigated the variation in Cr-CEST signal with temperature, from 35 °C to 39 °C (Extended Data Fig. 7) as the temperature change in BAT due to cold exposure was expected to be within 2 °C^50–52^. Extended Data Fig. 7 shows that only when the concentration of creatine was equal to or higher than 10 mM, the Cr-CEST signal showed an observable tendency to gradually decrease when temperature increased from 35 °C to 39 °C. With regard to the phantom data, we estimated that the in vivo creatine concentration in BAT is about 2.5 mM before cold exposure and about 5 mM after cold exposure. In addition, the dynamic Cr-CEST in WAT remained stable. All of this indicated that the increases in the BAT Cr-CEST signal due to drugs and cold exposure were not caused by temperature itself.

### B_0_ and B_1_ maps of rats and humans

**Extended Data Fig. 8:**
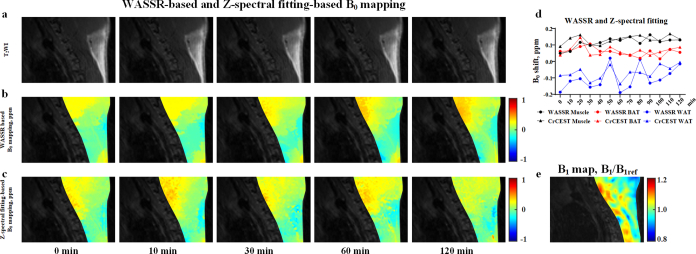
B_0_ and B_1_ mapping of the interscapular fat depot in rats. (**a-c**) Dynamic B_0_ mapping based on WASSR and Z-spectral fitting of fat depot before and post the injection of CL 316, 243 in rats, representative B_0_ maps (from n = 3). (**d**) The dynamics of WASSR-based and Z-spectral fitting-based B_0_ shift in muscle, BAT, and WAT during the 2-hour CL 316, 243 stimulation. (**e**) A representative B_1_ map of the interscapular fat depot in rats, representative B_1_ map (from n = 3). Note that the area containing lung cavity is not shown due to sparse signal and artifacts. Source data

Before Z-spectral fitting, the B_0_ inhomogeneities of Z-spectra were carefully corrected on a pixel-by-pixel basis with regard to the water (0 ppm) and fat (−3.4 ppm) signals in this study. We constructed both Z-spectral fitting-based and WAter Saturation Shift Referencing (WASSR)-based B_0_ maps under different time points after CL (Extended Data Fig. 8a–d); the results showed that two sets of B_0_ maps were essentially the same and the difference was smaller than the variations between the dynamic scans.

**Extended Data Fig. 9:**
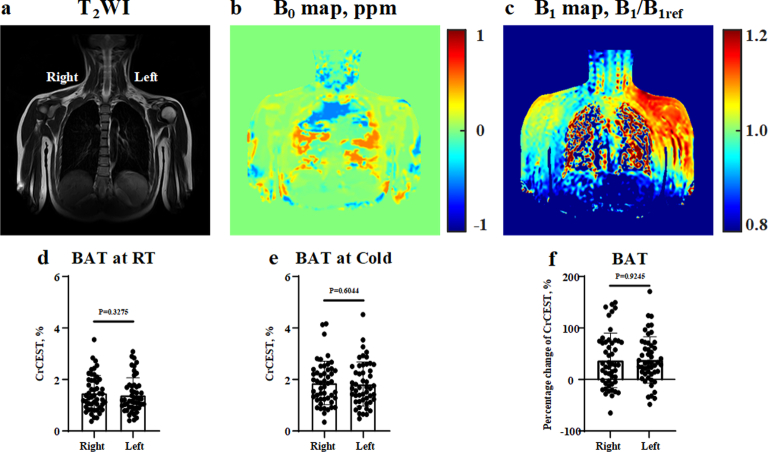
B_0_ and B_1_ inhomogeneity of human subjects did not affect the results of CrCEST MRI at 3T. (**a–c**) The representative T_2_WI and B_0_ and the B_1_ maps for the interscapular fat depot in human. (**d, e**) BAT CrCEST results showed no statistical differences between the left and right sides of the body before and after cold exposure (n = 50). (**f**) Percentage changes of CrCEST before and after cold exposure showed no statistical differences between the left and right sides (n = 50). RT, room temperature. Representative image (from n = 50). Data are presented as means ± s.d. Statistical analysis was performed using two-tailed paired Student’s t-tests. Source data

B_1_ correction for CEST studies is a common challenge in CEST MRI research. Luckily, the B_1_ maps of the adipose tissue and muscle areas in rats showed homogeneity, mostly within 10% variations (Extended Data Fig. 8e). For the human study using the 3T clinical MRI, B_0_ maps showed good homogeneity in the left and right sides of the body (Extended Data Fig. 9b). Although the B_1_ maps showed a difference between the two sides of the body (Extended Data Fig. 9c), the Cr-CEST signals before and after cold exposure and the corresponding Cr-CEST changes showed no statistically significant differences (Extended Data Fig. 9d–f), indicating that this range of B_0_ and B_1_ variations is acceptable.

### Cr-CEST normalized to the total or water signal

**Extended Data Fig. 10:**
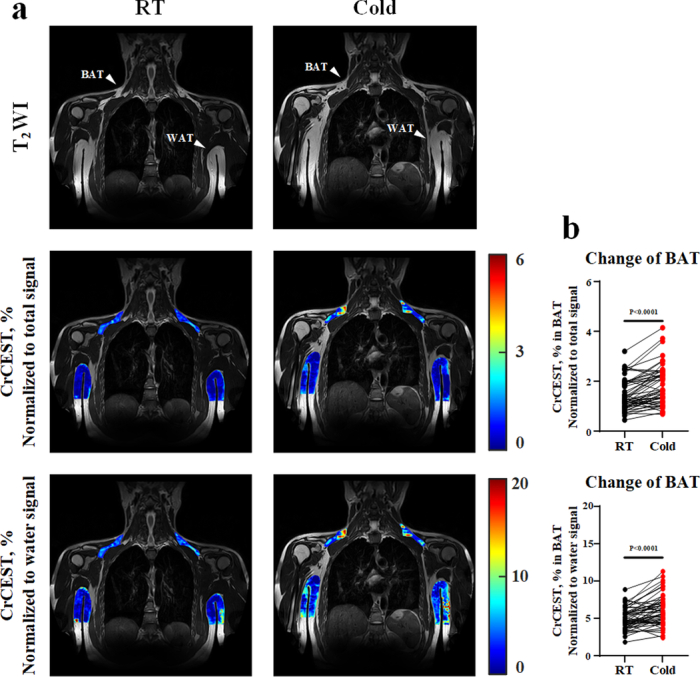
Normalized CrCEST maps of human subjects before and after cold exposure. (**a**) Besides rescaling the values, CrCEST maps normalized to the water signal did not change the overall spatial pattern of BAT activation. (**b**) Both types of normalized CrCEST showed significantly increased CrCEST due to cold exposure in humans. Statistical analysis was performed using two-tailed paired Student’s t-tests, n = 50 (**b**). Representative image (from n = 50). RT, room temperature. In T_2_WI, arrowheads were pointed to the BAT and WAT regions, respectively. Source data

By normalizing Cr-CEST to the total signal (the unsaturated signal at +300 ppm containing both water and fat signals) or the water signal (signal of the water peak) before and after cold exposure, Cr-CEST maps showed similar overall spatial patterns of BAT activation (Extended Data Fig. 10a) at different scales. Cr-CEST normalized to the water signal produced higher Cr-CEST values (5.01 ± 0.20% at room temperature and 6.16 ± 0.29% after cold exposure) than when normalized to the total signal (1.44 ± 0.09% at room temperature and 1.84 ± 0.11% after cold exposure), with a less relative cold-induced increment in BAT (25.81 ± 4.80% versus 33.45 ± 5.30%) (Extended Data Fig. 10b).

Normalization of Cr-CEST to the total signal represents the overall creatine concentration in tissue, whereas normalization to the water signal represents the creatine concentration in the intracellular fluid compartments. During the activation of BAT, the water signal increased while the lipid signal decreased. The FWF changed, while the total signal was relatively constant and was a better denominator than the water signal alone. Compared to the normalization of the total signal, the normalization of the water signal produced higher Cr-CEST values, but a lower relative cold-induced increment in BAT.

## Discussion

The principal physiological function of rodents and human BAT is thermogenesis. In the process of BAT thermogenesis, creatine, as one of the metabolites directly involved in ATP recycling, has an important role in ATP-based futile cycling. Creatine promotes the release of a molar excess of ADP from mitochondria^46^, which in turn promotes ATP regeneration, substrate oxidation and thermogenesis^13,20,53^. Therefore, we posited that creatine may serve as a biomarker for the metabolic function of adipose tissue. In this study, we demonstrated the feasibility of Cr-CEST MRI for noninvasive and functional imaging of adipose tissues to detect BAT activation in vivo. Dynamic Cr-CEST detected BAT adrenergic activation in rats in response to CL and NE injections or cold exposure, validated with [^18^F]FDG PET and CT and biochemical measurement of creatine concentration, as well as thermogenic gene expression and the monitoring of energy expenditure. Cr-CEST MRI also detected cold-induced BAT activation in humans, consistent with [^18^F]FDG PET and CT imaging.

CEST MRI of creatine kinase metabolites—creatine, phosphocreatine (PCr), ATP and ADP—was explored^54^ and displayed predominant CEST contrast from creatine at 2 ppm with negligible contributions from PCr, ATP and ADP. CEST detection of PCr is challenging and only feasible at the ultrahigh field from 9.4 to 21T^55–58^, probably because of its slow proton exchange rate (140 ± 60 per s)^54^. Unlike PCr, creatine usually has a relatively high exchange rate (950 ± 100 per s)^54^, making in vivo Cr-CEST more favourable. Cr-CEST MRI has a detection sensitivity a hundred times higher than ^1^H-MR spectroscopy (^1^H-MRS), enabling high-resolution imaging of creatine^37–39^. Cr-CEST MRI has been used to image several bioenergetic pathologies associated with tumour-bearing brains^35^, hearts^37^ and calf muscles^38,39^. In this study, we used Cr-CEST MRI for the functional imaging of fat tissues.

Activation of BAT thermogenesis with adrenergic receptor stimulation was accompanied by an increase in dynamic Cr-CEST contrast. We observed similar spatial distributions of Cr-CEST and FDG PET signals during BAT activation in rats due to CL or NE administration, indicating that high-resolution Cr-CEST contrast is consistent with FDG PET and CT imaging. By comparing the time course of dynamic signals, when the FDG signal readout typically requires 30–60 min of tracer uptake, the endogenous Cr-CEST MRI readout does not have such constraints. However, we observed larger variations in dynamic Cr-CEST signals than in FDG PET because of the inevitable motion artefacts in body MRI.

For Cr-CEST validation, in vivo ^1^H-MRS of creatine is a direct method. However, with high lipid content, adipose tissues have nine peaks on ^1^H-MRS (0.9, 1.3, 1.6, 2.02, 2.24, 2.75, 4.1, 4.3 and 5.3 ppm, respectively) when the water proton is at 4.7 ppm. Larger peaks shade the small creatine peak located at approximately 3.0 ppm. Suppression of both water and lipid signals for the quantification of creatine will also suppress the creatine signal, which makes in vivo ^1^H-MRS of creatine in adipose tissues impractical. Therefore, we validated Cr-CEST with ex vivo ^1^H-NMR experiments. To preserve metabolites, tissues were snap-frozen right after dissection. Next, samples were freeze-dried for at least 36 h in a SpeedVac before they were homogenized and centrifuged so that the lipid components were separated to enable the NMR of creatine.

NE activates 5′ AMP-activated protein kinase phosphorylation through the β_3-_adrenoreceptor to increase the glucose uptake of BAT^59,60^. BAT thermogenesis is activated after NE stimulation by the consumption of fatty acids and glucose^61^. Triglyceride breakdown provides fuel to support thermogenesis, regulated by UCP1 and UCP1-independent thermogenic mechanisms such as the futile creatine cycle^62^. However, our experiments in rats found that the effect due to 1.0 mg kg^−1^ of NE was not detectable with [^18^F]FDG PET as significant (*P* > 0.05) until the NE dosage was increased to 2.5 mg kg^−1^, which is consistent with previous studies^63,64^.

When mammals are exposed to cold, sympathetic nerves release NE, which activates brown adipocytes and induces thermogenesis^65^. In our experiments involving cold-stimulated rats and humans, BAT showed increased Cr-CEST contrast. Our in vivo human experiments before and after cold exposure also demonstrated the feasibility of the proposed Cr-CEST method to assess BAT activation in the context of clinical 3T applications. Many factors (for example, age, BMI) affect BAT activation^66^. The variation in baseline Cr-CEST in healthy adults in our study was significantly correlated to BMI, which is consistent with previous reports^66–68^.

In addition to BAT, beige adipocytes exhibit thermogenesis via the futile creatine cycle^18,19^. However, studying beige fat activation with the Cr-CEST we developed in this study requires higher detection sensitivity and spatial resolution, which will be the focus of our future work.

FDG PET imaging is based on the uptake of exogenous FDG, providing a readout of activated BAT. In contrast, a major advantage to using Cr-CEST is that as it provides endogenous imaging of tissue creatine, it allows BAT detection at rest and during activation. Therefore, unlike FDG PET, all volunteers showed positive Cr-CEST signals in BAT at baseline and after activation.

Of note, 50 volunteers were recruited for MRI to study baseline Cr-CEST variations dependent on BMI. Among these 50 healthy volunteers, 14 were randomly selected for PET and CT imaging. It is a limitation of this study that uneven numbers of individuals were recruited for MRI and PET. Thus, only a moderate correlation was found between Cr-CEST MRI and [^18^F]FDG PET. These data cannot be used to compare the detection sensitivity or statistical power between MRI and PET.

Currently, Dixon imaging and T2* mapping are the most commonly used MRI methods to differentiate BAT from WAT. Dixon imaging has an established track record for fat imaging and can differentiate BAT from WAT based on FWF^27,69^. T2* mapping has been exploited to differentiate between WAT and BAT based on the presence of iron in the mitochondria of BAT^70^. Because of the short imaging time, both methods usually have a high temporal and spatial resolution, making them incomparable for both acute monitoring and volumetric measurement of three-dimensional (3D) imaging^71^. However, both Dixon imaging and T2* mapping methods suffer from the ever-present sensitivity to phase wrapping and the inhomogeneities of the static B_0_ field^72^. More importantly, Dixon imaging provides mainly BAT mass-based information that is insensitive to adipocyte metabolic function^73^. The Cr-CEST contrast fitted from the whole Z-spectrum is inherently insensitive to phase wrapping and field inhomogeneity compared with Dixon imaging and T2* mapping. CEST Z-spectrum MRI can simultaneously obtain the FWF^74^ and creatine-mediated BAT function through Z-spectral fitting, realizing the combination of fat content and adipocyte metabolic function imaging.

The inevitable partial volume contamination of BAT by WAT and other ‘fat’ components (‘browning WAT’ and ‘beige adipocytes’) is a common challenge for MRI and PET imaging. However, the metabolic activity of adipose tissues measured with Cr-CEST (%) is a physiological parameter that is independent of tissue composition. Higher Cr-CEST means higher metabolic activity across all adipose tissue types in a general sequence of BAT > beige adipocytes > browning WAT > WAT. We further demonstrated that by increasing the spatial resolution of Cr-CEST, the partial volume effect can be reduced and the tissue boundaries are clearer. Furthermore, we also tried multi-slice Cr-CEST imaging in the rat interscapular BAT; the Cr-CEST signal of the whole BAT increased significantly after pharmacological stimulation.

As a limitation, the acquisition time for Cr-CEST imaging based on the full Z-spectral acquisition may prevent the capture of rapid creatine changes because of acute BAT activation. Recent studies demonstrated rapid 3D acquisition of CEST imaging by using multitasking^75^, multi-slice^76^ and snapshot^77^ acquisition. Hence, it may be feasible to capture rapid changes in creatine signals and 3D volumetric measurements of metabolically active BAT by incorporating these fast imaging sequences.

The duration and severity of obesity are positively associated with incident metabolic syndrome^78–80^, including T2D and cardiometabolic diseases. Early detection of the severity and complications of metabolic syndrome through metabolic or functional imaging of adipose tissues will be useful to prescribe effective interventions and lifestyle changes^81–84^. MRI offers the advantage of no radiation exposure compared to PET and CT and has gained popularity in the management of clinical obesity, especially for patients with T2D who cannot undergo [^18^F]FDG PET and CT imaging^85,86^. We demonstrated the feasibility of the endogenous metabolic Cr-CEST MRI technique in mapping BAT activity in rodents and humans. Endogenous Cr-CEST MRI may provide molecular insight into the pathogenesis, help with early detection and risk stratification, and serve as a biomarker for developing, evaluating and guiding new treatment strategies for metabolic diseases, using longitudinal and noninvasive means. Treatment evaluation and monitoring with the advanced Cr-CEST MRI may greatly enhance treatment efficacy and therefore reduce the socio-economic costs due to obesity.

## Methods

### Animal experiments

All animal experiments were approved by the Institutional Animal Use and Care Committee of Guangdong Provincial People’s Hospital. Sprague Dawley rats (male, 8 weeks old, 250–300 g) were obtained from Guangdong Medical Laboratory Animal Center. Rats were housed at 23 °C with free access to water and food under a 12/12 h light–dark cycle.

### In vivo 7T rat MRI

The rat MRI experiments were performed using a 7T small-bore MRI scanner (Bruker Biospec). Briefly, after fasted overnight, rats were anaesthetized (induced by 3.0% isoflurane, maintained with 1.5–2.0% isoflurane) and scanned in a supine position. A water-heated pad was used to maintain the body temperature of rats at 37 °C, and a balloon pressure sensor was placed on the abdomen for respiratory gating. Continuous physiological monitoring (rectal temperature, respiratory rate) was performed throughout the experiments using an MRI-compatible small-animal monitoring system (Small Animal Instruments).

Cr-CEST MRI was used to dynamically image interscapular fat every 10 min for up to 2 h after intraperitoneal injection of saline, NE or β_3_-adrenergic receptor agonists (CL, 1.0 mg kg^−1^ or 2.5 mg kg^−1^ body weight, Sigma-Aldrich). For the cold exposure experiment, rats were singly placed in individual precooled cages without bedding at 4 °C for 2 h in climate-controlled cold rooms. Cr-CEST MRI was performed on rats at room temperature and under cold exposure.

Cr-CEST Z-spectra were acquired from the central slice of the interscapular fat depot in rats with a pre-saturation pulse of 1 μT for 3 s (unless specified otherwise), followed by a rapid acquisition with relaxation enhancement (RARE) readout. Each Z-spectrum contained 54 images at varied frequency offsets, from −6 to 6 ppm at step-sizes of 0.25, ±8, ±10; +300 ppm was used as the reference image. The other imaging parameters were echo time (TE) = 3.6 ms; repetition time (TR) = 8,000 ms; field of view (FOV) = 40 × 40 mm^2^; matrix size = 100 × 100; in-plane resolution = 0.4 × 0.4 mm^2^; slice thickness = 1.5 mm; number of averages = 1; RARE factor = 36; and total acquisition time = 7 min 12 s.

To investigate the influence of spatial resolution, high-resolution Cr-CEST images were acquired similarly except for matrix size = 256 × 256; in-plane resolution = 0.156 × 0.156 mm^2^; number of averages = 2; TE = 4 ms; TR = 5,000 ms; 24 offsets (±10, ±7.5, ±5, ±4, ±3.5, ±3, ±2, ±1, ±0.5, 0, +2.5, +2.25, +1.75, +1.5 and +300 ppm); and acquisition time = 16 min. Low-resolution Cr-CEST was acquired with a matrix size = 128 × 128; in-plane resolution = 0.31 × 0.31 mm^2^; number of averages = 2; and acquisition time = 8 min. Multi-slice Z-spectra were acquired from three rats similarly, with 11 slices covering the entire interscapular BAT with TR = 3.2 s and a total acquisition time of 14 min.

All image processing and data analysis was performed in MATLAB (v.R2020a, MathWorks). Raw Z-spectral images (M_z_) were normalized with the reference image at +300 ppm (M_0_), carefully corrected for B_0_ inhomogeneities on a pixel-by-pixel basis in reference to water (0 ppm) or fat (−3.4 ppm) signal in fat-dominant tissues (the process of Z-spectral fitting further fine-tunes the water or fat resonances), reversed to 1-M_z_/M_0_ and then fitted to multi-Lorentzian functions containing the direct saturation of water and the main methylene fat, Cr-CEST, APT and broadband MT spectrum according to equations (1) and (2):

1 $$Z\left(\omega \right)=\mathop{\sum }\limits_{i=1}^{n}{L}_{i}(\omega )$$

2 $$L\left(\omega \right)=\frac{A}{1+4{\left(\frac{\omega -{\omega }_{0}}{l\omega }\right)}^{2}}$$

where *A*, *ω*_0_ and *lω* are the amplitude, centre frequency offset and line width of each peak, respectively.

The values and parameters for the first and second rounds of fittings are listed in Table 1. Three dominant components, including the direct saturation of bulk water, the main methylene fat and the MT peaks, were subtracted from the raw Z-spectrum and underwent a secondary fitting for the remaining two small peaks of Cr-CEST and APT. Motion artefacts during the 2 h Cr-CEST data acquisition were corrected by MATLAB’s intensity-based image registration routine imregister. Cr-CEST signals were averaged within the regions of interest (ROIs) of the central area of the interscapular BAT and WAT in reference to the corresponding T2-weighted images.

**Table 1:** Initial values and fitting constraints for the frequency offsets, line widths and amplitudes of the five main components contributing to Z-spectra in both rats and humans under all conditions

	Initial values (lower and upper limit)				
Parameters	Water	Fat or lipid	Semi-solid MT	Cr-CEST^a^	APT^a^
Frequency offset, ppm	0 (−0.5 to 0.5)	−3.4 (−4.0 to −3.0)	−1.5 (−4 to −1)	2.0 (1.6 to 2.4)	3.5 (3.0 to 4.0)
Line width, ppm	2.0 (0.1 to 10)	1.0 (0.1 to 5)	50 (20 to 100)	0.5 (0.1 to 5)	0.5 (0.1 to 5)
Amplitude, %	40 (0.01 to 100)	15 (0.01 to 100)	10 (0.01 to 40)	2 (0.01 to 20)	2 (0.01 to 20)

^a^Cr-CEST and APT peaks were refitted in a secondary fitting procedure after removing the three major signals of water, fat and MT.

### In vivo [^18^F]FDG PET and CT rat imaging

The rat [^18^F]FDG PET and CT experiments were performed using a Micro-PET/CT system (IRIS PET/CT, Inviscan). Briefly, after fasting for 8 h (required for PET studies), each rat was anaesthetized (induced by 3.0% isoflurane and maintained with 1.5–2.0% isoflurane) and placed onto the imaging bed in a supine position. A heated constant temperature airflow was used to maintain body temperature at 37 °C, and a balloon pressure sensor was placed on the abdomen for respiratory gating. Continuous physiological monitoring (rectal temperature, respiratory rate) was performed throughout the experiments using a small-animal monitoring system (Équipement Vétérinaire MINERVE).

Saline, NE (1.0 mg kg^−1^ and 2.5 mg kg^−1^), and CL (1.0 mg kg^−1^) were administered via intraperitoneal injection; at the same time, an intravenous injection of [^18^F]FDG (approximately 500 μCi) was administered via the rat’s lateral tail vein. Dynamic PET data were acquired to detect the glucose uptake of BAT for up to 2 h after an injection of either saline, NE or CL. After the PET scan, CT acquisition was performed at 80 kV and 0.9 mA, with a total acquisition time of 20 s. For PET quantification, the SUV_mean_ of interscapular BAT and WAT was measured in manually drawn ROIs in reference to the CT anatomical images.

### Human studies

The protocol for the human studies was approved by the Institutional Review Board of Guangdong Provincial People’s Hospital (no. KY-Q-2022–102–06). All participants provided written informed consent before the experiment. Fifty young and healthy volunteers (27 male, 23 female; age = 22.42 ± 1.85, BMI = 21.31 ± 3.51, mean ± s.d.) were recruited for Cr-CEST imaging of BAT activation at room temperature and 120 min after cold exposure. [^18^F]FDG PET and CT imaging was performed with a Biograph mCT Flow 64 PET/CT system (Siemens Healthcare) on 14 of the participants at room temperature and after 120 min of mild cold exposure.

Before each MRI or PET and CT imaging session, individuals were required to refrain from alcohol, caffeine, medication and exercise for the previous 48 h and to fast overnight (>8 h). The study flowchart is shown in Fig. 5. Briefly, on the first day, Cr-CEST imaging was performed in a 3T whole-body MRI scanner (Ingenia CX, Philips Healthcare) with a 32-channel surface receiving coil on the volunteers’ interscapular BAT region at room temperature (25 °C). Then, volunteers were transferred to an room temperature of 16–17 °C and wore a 6–10 °C cooling vest with ice packs for 120 min (changed every 30 min) to collect the cold-exposed Cr-CEST data.

For Cr-CEST imaging, the parameters of the turbo spin-echo-CEST sequence remained the same as in the in vivo rat experiments except for TE = 6.9 ms; TR = 6,000 ms; FOV = 500 × 500 mm^2^; reconstructed matrix size = 512 × 512; slice thickness = 5 mm; echo train length = 156; and total acquisition time = 5 min 30 s. The Cr-CEST post-processing procedure in the human studies was consistent with that in the rat studies. BAT and WAT Cr-CEST signals were quantified by drawing the ROI on the central area of the interscapular and subcutaneous abdominal, respectively, using T2-weighted images as anatomical references.

The PET and CT studies of 14 participants (age = 22.00 ± 1.47; sex = 7 male and 7 female; BMI = 20.67 ± 2.05, mean ± s.d.) were performed 2 weeks after the MRI. CT scans were performed at 120 kV, 230 mAs and 3-mm thickness of layers, followed by a PET scan. For the PET and CT studies at RT, 5 mCi [^18^F]FDG was injected intravenously into each participant. The PET and CT scans were acquired 60 min later. Two weeks later, volunteers were invited back for a second PET and CT scan after being exposed to the cold and wore a 6–10 °C cooling vest with ice packs for 60 min (changed every 30 min). During cold exposure, individuals wore shorts and T-shirts for sedentary activities, such as reading a book and watching TV, and were instructed to avoid physical activity. 5 mCi of [^18^F]FDG was injected intravenously into each volunteer. After another 60 min of cold exposure with the cooling vest on, a PET and CT was performed. The SUV_mean_ of the central area of the interscapular BAT and subcutaneous abdominal WAT was measured in manually drawn ROIs according to the BARCIST standard^23^ in reference to the CT anatomical images (Hounsfield units were between −190 and −10, avoiding the glands, blood vessels, skeletal muscles and air spaces). Two different operators (both with more than 3 years of experience in BAT imaging research) delineated the ROI separately and showed a good interclass correlation coefficient of 0.90 (*P* < 0.0001).

### Phantom preparation and MRI

Phantoms (*n* = 3 sets) contained vials with creatine concentrations of 2.5 mM, 5.0 mM, 7.5 mM, 10 mM, 15 mM, and 20 mM in PBS (pH 7.0). The temperature of the phantom was controlled with a hot-water pad and monitored with a temperature probe; the corresponding Cr-CEST MRI data were collected at 35 °C, 37 °C and 39 °C. Cr-CEST Z-spectra were acquired from the central slice of the phantom with a pre-saturation pulse of 1 μT for 3 s, followed by a RARE readout. Each Z-spectrum contained 54 images at varied frequency offsets containing from −6 to 6 ppm at step-sizes of 0.25 ppm, ±8, ±10, with +300 ppm used as the reference image. The other imaging parameters were TE = 3.6 ms; TR = 6,000 ms; FOV = 40 × 40 mm^2^; matrix size = 160 × 160; in-plane resolution = 0.25 × 0.25 mm^2^; slice thickness = 1.0 mm; number of averages = 1; RARE factor = 89; and total acquisition time = 5 min 24 s.

### B_0_ field maps

For Z-spectral fitting-based B_0_ mapping, B_0_ inhomogeneities from the Cr-CEST data were corrected with reference to the water (0 ppm) or fat (−3.4 ppm) signal in fat-dominant tissues. The process of Z-spectral fitting further fine-tunes the water or fat resonance.

For WASSR-based B_0_ mapping, the B_0_ field map was defined as the offset of the water peak in the Z-spectra with reference to 0 ppm. WASSR data from rats were acquired with a pre-saturation pulse of 0.5 μT for 500 ms followed by a RARE readout. Each Z-spectrum contained 42 images at varied frequency offsets containing from −2 to 2 ppm at step-sizes of 0.1 ppm; +300 ppm was used as the reference image. The other imaging parameters were TE = 3.6 ms; TR = 1,500 ms; FOV = 40 × 40 mm^2^; matrix size = 160 × 160; in-plane resolution = 0.25 × 0.25 mm^2^; slice thickness = 1.5 mm; number of averages = 1; RARE factor = 89; and total acquisition time = 1 min 3 s.

### B_1_ field maps

B_1_ field maps from rats were obtained using a two-dimensional single-slice RARE readout sequence with TE = 10 ms; TR = 10,000 ms; FOV = 40 × 40 mm^2^; and matrix size = 80 × 80. Two images were obtained using preparation pulses with 60° and 120° flip angles. The actual flip angle (θ) for each pixel was calculated as shown in equation (3).

3 $$\frac{{\mathrm{cos }}({\mathrm{2}}{{\theta}})}{{\mathrm{cos}}({{\theta }})}=\frac{{\mathrm{I}}({\mathrm{120}}^\circ )}{{\mathrm{I}}({\mathrm{60}}^\circ )}$$

where I (60°) and I (120°) denote pixel signals with 60° and 120° preparation flip angles, respectively. The relative B_1_ map was constructed as *θ*/60° for each pixel.

B_1_ field maps from humans were obtained similarly except for TE = 50 ms; TR = 6,000 ms; FOV = 500 × 500 mm^2^; and matrix size = 400 × 400.

### Gene expression analysis (quantitative PCR with reverse transcription)

Total RNA was extracted from frozen tissue using the TRNzol Universal Reagent (TIANGEN Biotech) and quantified using a NanoDrop 2000 ultraviolet-visible spectrophotometer (Thermo Fisher Scientific). Using quantitative PCR with reverse transcription (RT–qPCR) and the PrimeScript RT Reagent Kit (Takara Bio), complementary DNA (cDNA) was prepared using 1 µg total RNA. RT–qPCR was performed on cDNA using the TB Green Premix EX Taq II kit (Takara Bio) and the CFX96 Real-Time PCR Detection System (Bio-Rad Laboratories). Normalized mRNA expression was calculated with the $2^{-\Delta\Delta{\mathrm{C}}_{\mathrm{T}}}$ method, using vinculin (VCL) mRNA as the reference. Primer sequences used for the RT–qPCR of the rat samples are as follows: *Pparg1α* (forward ACACATCGCAATTCTCCCTTGTA; reverse CTTTCAGACTCCCGCTTCTCATA); *Ckb* (forward AAGACTGACCTCAACCCAGACAA; reverse CTTCTA CTGCCAGCTTCTCGATG); *Dio2* (forward GCCAACGTAGCTTATGGGGT; reverse TTCTCCAGC CAACTTCGGAC); *Ucp1* (forward TCTACGATACGGTCCAAGAGTACT; reverse AAGCATTGTAGGTCCCAGTGTAG); *Vcl* (forward CTGACCTCCTGCTTACCTTTGAT; reverse GCAGCTCTTTGACAGTGTTCATT).

### ^1^H-NMR spectroscopy

Brown fat tissue samples (approximately 200 mg) were freeze-dried for at least 36 h in a SpeedVac. Dehydrated tissue was homogenized in PBS buffer, pH 7.4, and centrifuged at 13,000*g* for 10 min at 4 °C; the soluble liquid below the fat cake was transferred to a new tube after centrifugation. The supernatant was reconstituted in a 600 μl mixture containing 550 μl PBS buffer and 50 μl tetramethylsilane (TMS) in D_2_O (99.9% D_2_O + 0.05% TMS v/v), vortex-mixed for 60 s and transferred to 5-mm NMR tubes. The ^1^H-NMR spectra were acquired with a Bruker 600 MHz Avance III HD spectrometer (Bruker Biospec). ^1^H-NMR spectra were recorded using a one-dimensional CPMG pulse sequence; the acquisition parameters were: number of scans 64; 90° pulse width, 11.4 μs; acquisition time 2.73 s; and relaxation delay 4.0 s. The spectrum was then phase-adjusted manually, followed by baseline correction and frequency alignment with reference to the 0 ppm TMS signal using MestReNova v.14.0 (Mestrelab Research). Metabolites were identified and quantified by fitting the spectral lines of the library compounds into the recorded ^1^H-NMR spectrum of the tissue extract. Absolute quantification was based on the peak amplitude of the TMS signal. Metabolite concentrations are exported as mmol l^−1^ in the NMR sample.

### Metabolic cages

Rats were fasted overnight and single-housed in metabolic chambers (Promethion high-definition behavioural phenotyping system, Sable Systems International) for respiratory monitoring. After measuring the basic metabolic rate for 30 min without interruption. Rats were injected intraperitoneally with saline, NE or CL at the dose of 1.0 mg kg^−1^ body weight; oxygen consumption (VO_2_) was recorded every 3 min for 2 h using the Sable Systems data acquisition software. Data were parsed and analysed using the Sable Systems International Macro Interpreter software (v.22.10) using the One Click Macro (v.2.51.4).

### Statistical analysis

Sample numbers, the number of biological replicates and the statistical analysis methods used are provided in the figure legends. Statistical analysis was performed using Prism v.8.3.0 (GraphPad Software). All statistical tests are fully described in the figure legends and meet the criteria for a normal distribution with similar variance. Two-tailed Student’s *t*-tests were used for comparisons between two groups. Data are presented as the mean ± s.e.m. unless otherwise stated. *P* < 0.05 was deemed statistically significant.

### Reporting summary

Further information on research design is available in the Nature Portfolio Reporting Summary linked to this article.

### Supplementary information

Reporting Summary

### Source data

Source Data Fig. 2 Statistical source data.

Source Data Fig. 3 Statistical source data.

Source Data Fig. 4 Statistical source data.

Source Data Fig. 6 Statistical source data.

Source Data Extended Data Fig. 1 Statistical source data.

Source Data Extended Data Fig. 2 Statistical source data.

Source Data Extended Data Fig. 5 Statistical source data.

Source Data Extended Data Fig. 7 Statistical source data.

Source Data Extended Data Fig. 8 Statistical source data.

Source Data Extended Data Fig. 9 Statistical source data.

Source Data Extended Data Fig. 10 Statistical source data.

## Data Availability

All source data are provided as Supplementary Information. Source data are provided with this paper.
