# Supplementary material for: Non-invasive mapping of brown adipose tissue activity with magnetic resonance imaging
Source: Nat Metab. 2024 Jul 25;6(7):1367–79. doi: 10.1038/s42255-024-01082-z (PMC11272596; doi:10.1038/s42255-024-01082-z)
Supplement: Supplementary file 1 — Reporting Summary [file 42255_2024_1082_MOESM1_ESM.pdf]

nature portfolio

Corresponding author(s): Zaiyi Liu

Last updated by author(s): May 10, 2024

Reporting Summary

Nature Portfolio wishes to improve the reproducibility of the work that we publish. This form provides structure for consistency and transparency in reporting. For further information on Nature Portfolio policies, see our Editorial Policies and the Editorial Policy Checklist.

Statistics

For all statistical analyses, confirm that the following items are present in the figure legend, table legend, main text, or Methods section.

n/a	Confirmed	
☐	☒	The exact sample size (*n*) for each experimental group/condition, given as a discrete number and unit of measurement
☐	☒	A statement on whether measurements were taken from distinct samples or whether the same sample was measured repeatedly
☐	☒	The statistical test(s) used AND whether they are one- or two-sided<br>*Only common tests should be described solely by name; describe more complex techniques in the Methods section.*
☒	☐	A description of all covariates tested
☒	☐	A description of any assumptions or corrections, such as tests of normality and adjustment for multiple comparisons
☐	☒	A full description of the statistical parameters including central tendency (e.g. means) or other basic estimates (e.g. regression coefficient) AND variation (e.g. standard deviation) or associated estimates of uncertainty (e.g. confidence intervals)
☐	☒	For null hypothesis testing, the test statistic (e.g. *F*, *t*, *r*) with confidence intervals, effect sizes, degrees of freedom and *P* value noted<br>*Give P values as exact values whenever suitable.*
☒	☐	For Bayesian analysis, information on the choice of priors and Markov chain Monte Carlo settings
☒	☐	For hierarchical and complex designs, identification of the appropriate level for tests and full reporting of outcomes
☒	☐	Estimates of effect sizes (e.g. Cohen's *d*, Pearson's *r*), indicating how they were calculated

Our web collection on statistics for biologists contains articles on many of the points above.

Software and code

Policy information about availability of computer code

Data collection	-Male rat MRI experiments were performed using a 7T small-bore MRI scanner (Bruker Biospec, Billerica, MA, USA).<br>-MRI-compatible small animal-monitoring system (Small Animal Instruments, Stony Brook, NY, USA)<br>-Male rat 18F-FDG PET/CT experiments were performed using a Micro-PET/CT system (IRIS PET/CT, Inviscan, Strasbourg, France).<br>-Small-animal-monitoring system (Equipement Veterinaire Minerve, Esternay, France).<br>-Human MRI imaging was performed with a 3T whole-body MRI scanner (Ingenia CX, Philips Healthcare, Best, the Netherlands).<br>-Human 18F-FDG PET/CT imaging was performed with a Biograph mCT Flow 64 PET/CT system (Siemens Healthcare, Erlangen, Germany).<br>-Quantitative PCR was performed with the CFX96 Real-Time PCR Detection System (Bio-Rad Laboratories, Inc, Hercules, California, USA).<br>-The 1H-NMR spectra were acquired using a Bruker 600 MHz Avance III HD spectrometer (Bruker Biospec, Billerica, MA, USA).<br>-Promethion high-definition behavioral phenotyping system (Sable Systems International, Las Vegas, NV, USA).
Data analysis	GraphPad Prism (v.8.3.0); Sable Systems International Macro Interpreter software (v.22.10); One-Click Macro (v2.51.4); MestReNova (V14.0, Mestrelab Research, Spain) software; MATLAB (R2020a, MathWorks, Natick, MA).

For manuscripts utilizing custom algorithms or software that are central to the research but not yet described in published literature, software must be made available to editors and reviewers. We strongly encourage code deposition in a community repository (e.g. GitHub). See the Nature Portfolio guidelines for submitting code & software for further information.

Data

Policy information about availability of data

All manuscripts must include a data availability statement. This statement should provide the following information, where applicable:
- Accession codes, unique identifiers, or web links for publicly available datasets
- A description of any restrictions on data availability
- For clinical datasets or third party data, please ensure that the statement adheres to our policy

All source data are provided as supplementary information.

Human research participants

Policy information about studies involving human research participants and Sex and Gender in Research.

Reporting on sex and gender	Twenty-seven male and twenty-three female volunteers were equally included based on willingness to participate in this study.
Population characteristics	Fifty young and healthy volunteers (27 male, 23 female, Age: 22.42 ± 1.85, BMI: 21.31 ± 3.51, mean ± s.d) were recruited in this study.
Recruitment	The healthy volunteers were recruited using the call for trial participation by the group chat of Southern Medical University. No self-selection bias during the recruitment in this study.
Ethics oversight	Informed consent was obtained from all participants. All human studies were approved by the Ethical Review Committee in the Guangdong Provincial People's Hospital, China.

Note that full information on the approval of the study protocol must also be provided in the manuscript.

Field-specific reporting

Please select the one below that is the best fit for your research. If you are not sure, read the appropriate sections before making your selection.

☒ Life sciences ☐ Behavioural & social sciences ☐ Ecological, evolutionary & environmental sciences

For a reference copy of the document with all sections, see nature.com/documents/nr-reporting-summary-flat.pdf

Life sciences study design

All studies must disclose on these points even when the disclosure is negative.

Sample size	No sample size predetermination was performed. The sample size was chosen based on previous experience to achieve statistical significance.
Data exclusions	No data were excluded.
Replication	All experiments have been successfully replicated showing the same results for at least three times.
Randomization	All subjects were randomly allocated into the study groups.
Blinding	All experimental data were reviewed blind by at least two independent researchers.

Reporting for specific materials, systems and methods

We require information from authors about some types of materials, experimental systems and methods used in many studies. Here, indicate whether each material, system or method listed is relevant to your study. If you are not sure if a list item applies to your research, read the appropriate section before selecting a response.

Materials & experimental systems

n/a	Involved in the study
☒	☐ Antibodies
☒	☐ Eukaryotic cell lines
☒	☐ Palaeontology and archaeology
☐	☒ Animals and other organisms
☐	☒ Clinical data
☒	☐ Dual use research of concern

Methods

n/a	Involved in the study
☒	☐ ChIP-seq
☒	☐ Flow cytometry
☒	☐ MRI-based neuroimaging

Animals and other research organisms

Policy information about studies involving animals; ARRIVE guidelines recommended for reporting animal research, and Sex and Gender in Research

Laboratory animals	Sprague Dawley rats, male, age 8 weeks.
Wild animals	The study did not use wild animals.
Reporting on sex	This initial technological development is not expected to be gender-related. In addition, obesity is common in both male and female.
Field-collected samples	The study did not use field-collected samples.
Ethics oversight	All animal experiments were approved by the Institutional Animal Use and Care Committee of Guangdong Provincial People's Hospital.

Note that full information on the approval of the study protocol must also be provided in the manuscript.

Clinical data

Policy information about clinical studies
All manuscripts should comply with the ICMJE guidelines for publication of clinical research and a completed CONSORT checklist must be included with all submissions.

Clinical trial registration	Not applicable.
Study protocol	Proof-of-concept and technical-development study.
Data collection	All the healthy volunteers were recruited from September 2021 to January 2024 at Southern Medical University in this study. All the information about human participants has been provided in the manuscript.
Outcomes	Not applicable.
